# Monolithic integrated micro-supercapacitors with ultra-high systemic volumetric performance and areal output voltage

**DOI:** 10.1093/nsr/nwac271

**Published:** 2022-11-26

**Authors:** Sen Wang, Linmei Li, Shuanghao Zheng, Pratteek Das, Xiaoyu Shi, Jiaxin Ma, Yu Liu, Yuanyuan Zhu, Yao Lu, Zhong-Shuai Wu, Hui-Ming Cheng

**Affiliations:** State Key Laboratory of Catalysis, Dalian Institute of Chemical Physics, Chinese Academy of Sciences, Dalian 116023, China; Department of Biotechnology, Dalian Institute of Chemical Physics, Chinese Academy of Sciences, Dalian 116023, China; State Key Laboratory of Catalysis, Dalian Institute of Chemical Physics, Chinese Academy of Sciences, Dalian 116023, China; Dalian National Laboratory for Clean Energy, Chinese Academy of Sciences, Dalian 116023, China; State Key Laboratory of Catalysis, Dalian Institute of Chemical Physics, Chinese Academy of Sciences, Dalian 116023, China; University of Chinese Academy of Sciences, Beijing 100049, China; State Key Laboratory of Catalysis, Dalian Institute of Chemical Physics, Chinese Academy of Sciences, Dalian 116023, China; State Key Laboratory of Catalysis, Dalian Institute of Chemical Physics, Chinese Academy of Sciences, Dalian 116023, China; University of Chinese Academy of Sciences, Beijing 100049, China; State Key Laboratory of Catalysis, Dalian Institute of Chemical Physics, Chinese Academy of Sciences, Dalian 116023, China; State Key Laboratory of Catalysis, Dalian Institute of Chemical Physics, Chinese Academy of Sciences, Dalian 116023, China; Department of Biotechnology, Dalian Institute of Chemical Physics, Chinese Academy of Sciences, Dalian 116023, China; State Key Laboratory of Catalysis, Dalian Institute of Chemical Physics, Chinese Academy of Sciences, Dalian 116023, China; Dalian National Laboratory for Clean Energy, Chinese Academy of Sciences, Dalian 116023, China; Faculty of Materials Science and Engineering/Institute of Technology for Carbon Neutrality, Shenzhen Institute of Advanced Technology, Chinese Academy of Sciences, Shenzhen 518055, China; Shenyang National Laboratory for Materials Science, Institute of Metal Research, Chinese Academy of Sciences, Shenyang 110016, China; Advanced Technology Institute, University of Surrey, Guildford GU2 7XH, UK

**Keywords:** monolithic, integrated micro-supercapacitors, areal output voltage, lithography, MXene

## Abstract

Monolithic integrated micro-supercapacitors (MIMSCs) with high systemic performance and cell-number density are important for miniaturized electronics to empower the Internet of Things. However, fabrication of customizable MIMSCs in an extremely small space remains a huge challenge considering key factors such as materials selection, electrolyte confinement, microfabrication and device-performance uniformity. Here, we develop a universal and large-throughput microfabrication strategy to address all these issues by combining multistep lithographic patterning, spray printing of MXene microelectrodes and controllable 3D printing of gel electrolytes. We achieve the monolithic integration of electrochemically isolated micro-supercapacitors in close proximity by leveraging high-resolution micropatterning techniques for microelectrode deposition and 3D printing for precise electrolyte deposition. Notably, the MIMSCs obtained demonstrate a high areal-number density of 28 cells cm^−2^ (340 cells on 3.5 × 3.5 cm^2^), a record areal output voltage of 75.6 V cm^−2^, an acceptable systemic volumetric energy density of 9.8 mWh cm^−3^ and an unprecedentedly high capacitance retention of 92% after 4000 cycles at an extremely high output voltage of 162 V. This work paves the way for monolithic integrated and microscopic energy-storage assemblies for powering future microelectronics.

## Teaser

This work developed the monolithic integrated micro-supercapacitors with an areal-number density of 28 cells cm^−2^, areal output voltage of 75.6 V cm^−2^, systemic volumetric energy density of 9.8 mWh cm^−3^ and excellent stability.

## INTRODUCTION

For truly realizing the Internet of Things consisting of miniaturized electronics, microscale electrochemical energy-storage systems with high systemic performance, superior cell-number density, tunable capacitance and output voltage are required [1–4]. In this regard, on-chip interdigitated micro-supercapacitors (MSCs) free of separators and external metal connection wires, simultaneously with reliable electrochemical performance and tunable connection, will greatly boost the cell-number density and systemic performance for monolithic integrated MSCs (MIMSCs) with desirable customizability in a limited space [5,6].

So far, enormous progress has been achieved for MSCs, including electrode materials engineering, high-precision microfabrication techniques, functional properties and their integration with microelectronic systems [7–9]. However, scalable production of fully-functioning compact MIMSCs with high systemic performance, superior cell-number density and tunable performance is still challenging, due to the difficulty in the precise deposition of electrolytes on densely-packed MSCs for electrochemical isolation, sacrifice in electrochemical performance during complex microfabrication procedures and limited performance uniformity among numerous individual cells in large-scale arrays. In particular, the footprint of a single cell and interspace between adjacent cells of most reported MIMSCs are too large, resulting in low systemic performance and cell-number density [10–12]. For instance, screen-printed MXene MIMSCs exhibited a low areal cell-number density of 1.7 cells cm^−2^ [13] and inkjet-printed MXene/PH1000 MIMSCs presented an areal cell-number density of 9 cells cm^−2^ [14]. Although some dense microelectrodes arrays have been constructed previously by lithography [15], focused ion beam patterning [16], laser scribing [17] and inkjet printing techniques [18,19], the functioning MIMSCs have not been achieved owing to the lack of an accurate electrolyte-deposition technology. Second, considerable attention should be paid to improving the electrochemical performance, which is usually sacrificed due to poor compatibility between electrode materials with multistep complex microfabrication procedures [20,21]. For example, MIMSCs fabricated via electrohydrodynamic jet printing demonstrated a high areal-number density of 54.9 cells cm^−2^. However, this delicate fabrication technique has severely limited the selection of materials, resulting in low areal capacitance of a single cell (127.5 μF cm^−2^) [22]. Third, the performance uniformity among numerous individual cells, including microelectrodes and electrolytes, determined by each step in the microfabrication processes, crucially affects the output voltage and cyclability of the resultant MIMSCs [23,24]. Therefore, innovation in the whole microfabrication technique to develop MIMSCs that simultaneously achieve superior cell-number density and stable systemic performance is highly necessary.

Herein, we develop a universal and reliable strategy to achieve mass production of microelectrodes with high cell-number density and precise deposition of gel electrolytes. This is achieved by combining multistep lithography for defining the micropatterns, spray printing of MXene microelectrodes and 3D printing of quasi-solid-state gel electrolytes. Each individual MXene-based MSC (M-MSC) exhibited an extremely small footprint of 0.018 cm^2^, high areal capacitance of 4.1 mF cm^−2^, volumetric capacitance of 457 F cm^−3^ and stable performance at ultra-high scan rate of ≤500 V s^−1^. A novel 3D printing process was developed to deposit the designed gel electrolyte ink, resulting in the electrochemical isolation of adjacent M-MSCs in the monolithic array of M-MSCs (denoted as M-MIMSCs) and demonstration of outstanding performance uniformity. The resultant M-MIMSCs offered an exceptional areal cell-number density of 28 cells cm^−2^ (340 cells on 3.5 × 3.5 cm^2^), systemic volumetric energy density of 9.8 mWh cm^−3^, ultra-high output voltage of 200 V and the highest areal output voltage of 75.6 V cm^−2^ so far. This work provides an avenue for achieving super-dense arrays of MSCs for powering diverse microelectronic applications.

## RESULTS AND DISCUSSION

### Fabrication of M-MIMSCs

**Figure 1.**
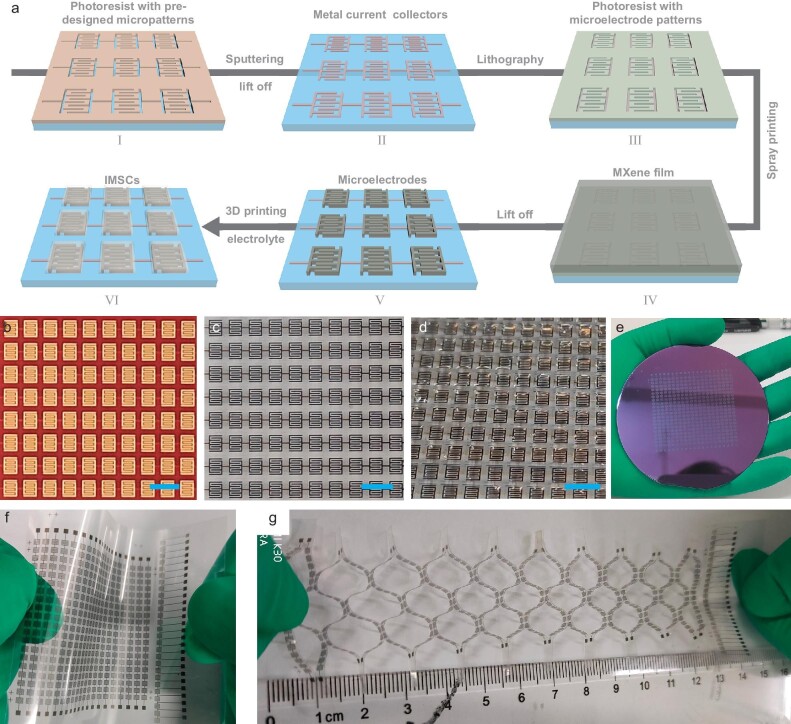
Monolithic lithography for compact and customizable M-MIMSCs. (a) Schematic of the fabrication of M-MIMSCs. (b–d) Optical images for the key steps of (III), (V) and (VI), scale bars: 3 mm. (e) Photograph of a 3-inch wafer-scale M-MIMSCs containing 400 cells. (f) Flexibility and (g) stretchability of M-MIMSCs on a flexible polyethylene terephthalate substrate.

**Figure 2.**
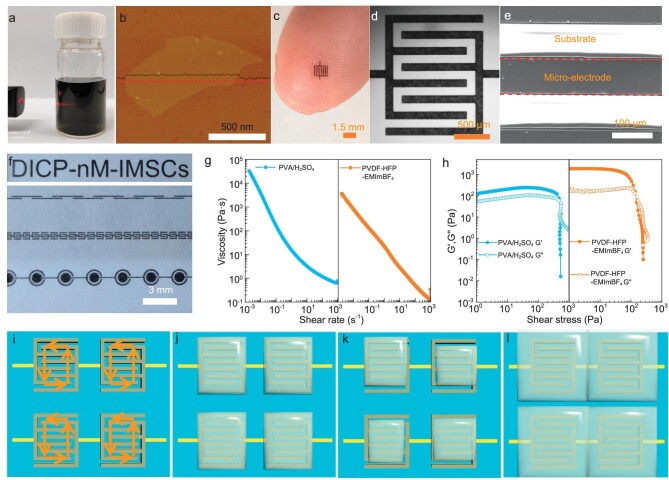
Characterization of M-MIMSCs. (a) Tyndall scattering profile of aqueous MXene dispersion. (b) Atomic force microscope image of MXene nanosheet. (c) Photograph of a M-MSC on a human finger. (d and e) SEM images of the interdigital microelectrodes. (f) Photographs of micropatterns with various shapes (from top to bottom: letters, linear, foldable and concentric circles). (g and h) Rheological properties of gel electrolyte inks for 3D printing. ((i–l) Schematic of 3D printing gel electrolyte in rectangle printing path (i); under optimal condition gel electrolyte precisely covering each cell (j); under unsuitable conditions, gel electrolyte incomplete coverage or uneven coverage the cells (k); and gel electrolyte contacted with each other for adjacent cells resulting in failure electrochemical isolation (l).

The on-chip microfabrication of M-MIMSCs is illustrated in Fig. 1 and Supplementary Fig. S1. First, a thin photoresist (AZ4620) layer with predesigned micropatterns on a target substrate (e.g. Si, glass or flexible polyethylene terephthalate, Supplementary Fig. S2) was obtained by using the lithographic process. Next, Au/Ti (250 nm) was sputtered over the substrate followed by lift-off in acetone, forming current collectors for microelectrodes and electrical connections between adjacent microcells (Supplementary Fig. S3). Then, another photoresist layer with microelectrode patterns was obtained by the same lithographic process on Au/Ti current collectors (Fig. 1b and Supplementary Fig. S4). In terms of the requirement for high-precision microelectrodes, an aqueous dispersion of small-size MXene (1-nm-thick nanosheet) with a lateral dimension of 100–600 nm (Fig. 2a and b, and Supplementary Fig. S5) was prepared by etching Ti_3_AlC_2_ with LiF and HCl [13,25], followed by sonication. This was chosen as the active material owing to its high capacitance, metallic conductivity and solution-processing ability [15]. Subsequently, a MXene film (Supplementary Fig. S6) was printed on the entire surface by spray printing. Afterward, the MXene film deposited on the unexposed photoresist regions was removed through another lift-off process in acetone, creating high-resolution interdigital microelectrodes with a fine feature size of 100 μm and an extremely small footprint of 0.018 cm^2^ (Fig. 2c–e). Satisfactorily, no residual active material was observed from the precise profile of the interdigital microelectrodes even after bath sonication used for the lift-off process (Fig. 2e), implying the strong adhesion between the metal surface and the MXene nanosheets. Further, 340 integrated M-MSCs with an adjacent interval of 600 μm were fabricated on a 3.5 × 3.5 cm^2^ rigid substrate, achieving an unprecedented areal-number density of 28 cells cm^−2^ that far exceeds values from previous reports [11,14,23,24], exhibiting application potential in real situations requiring compact integration energy supply (Fig. 1c and e, and Supplementary Fig. S7). Remarkably, M-MIMSCs with 340 cells fabricated on a flexible polyethylene terephthalate substrate weighed only 78 mg, which is critical for miniaturized robots unable to carry heavy power-supply units [26]. They also exhibited robust flexibility and stretchability (Fig. 1f and g). Micrometer-sized electrodes with various customizable shapes could be readily constructed using predesigned masks, demonstrating the aesthetic diversity of this strategy (Fig. 2h). Finally, to guarantee the electrochemical isolation of adjacent microcells in close proximity, printable quasi-solid-state gel electrolyte inks were prepared (Fig. 2g and h, and Supplementary Fig. S8) and then precisely deposited on microcells by using a reliable and controllable 3D printing technique (Figs 1d and 2j, Supplementary Fig. S9a and Supplementary Video S1). It is worth noting that the appropriate 3D printing mode is critical for the fabrication of high areal-cell-number-density and stable MIMSCs. As shown in Supplementary Fig. S10, under inappropriate printing modes, such as dot printing and line-segment printing, no matter how the printing parameters are adjusted, the gel electrolyte could not achieve complete coverage of the cells while ensuring the electrochemical isolation of adjacent microcells. Without isolation, multiple MSCs connected in series would act as a single MSC, so effective voltage regulation could not be achieved. To this end, precise deposition for polyvinyl alcohol/H_2_SO_4_ (PVA/H_2_SO_4_) gel electrolyte was achieved at an extrusion needle with inner diameter of 210 μm, a printing speed of 4 mm s^−1^ and an extrusion pressure of 25 psi under the appropriate rectangular printing path, enabling electrochemical isolation of each cell (Figs 1d and 2j, and Supplementary Fig. S9a), avoiding incomplete and uneven coverage (Fig. 2k and Supplementary Fig. S9b) and contact with each other (Fig. 2l and 9). Incomplete coverage of electrolyte on microcells resulted in an incomplete utilization of the microelectrodes and inferior systemic performance. Uneven coverage resulted in poor performance consistency of each microcell in the MIMSCs and hence poor cycle stability.

### Superior performance of an individual M-MSC

**Figure 3.**
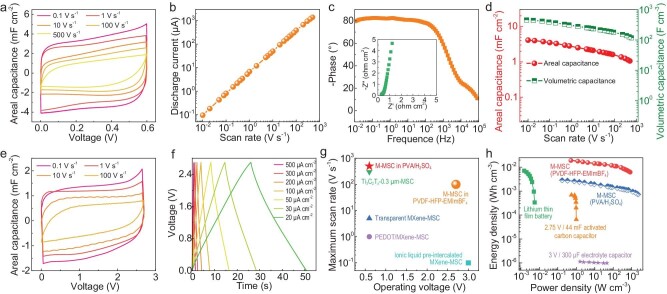
Electrochemical characterization of a single M-MSC in aqueous and ionic liquid-gel electrolytes. (a–d) CV curves over scan rates from 0.1 to 500 V s^−1^ (a), discharge current (values are taken at 0.3 V) as a function of scan rate (b), Bode phase angle plot (c) and areal capacitance and volumetric capacitance versus scan rate (d) of M-MSC in PVA/H_2_SO_4_ gel electrolyte. Inset in (c) shows corresponding Nyquist spectra. (e and f) CV curves measured at different scan rates of 0.1–100 V s^−1^ (e) and GCD profiles at current densities of 20–500 μA cm^−2^ (f) of M-MSC in PVDF-HFP-EMIMBF_4_ gel electrolyte. (g) Maximum scan rate versus the cell operating voltage of our M-MSCs compared with other MXene-based MSCs (Ti_3_C_2_T_x_-0.3 μm-MSC [15], ionic liquid pre-intercalated MXene MSC [27]. PEDOT/MXene MSC [28], transparent MXene MSC [29]). (h) Ragone plot of our M-MSCs with commercial energy-storage devices.

To demonstrate their superior performance, we evaluated an individual M-MSC with a footprint of 0.018 cm^2^ (microelectrode thickness of 90 nm) using a PVA/H_2_SO_4_ polymer gel electrolyte. As expected, approximately rectangular cyclic voltammetry (CV) curves measured at different scan rates (Fig. 3a) and nearly linear symmetric triangular-shaped galvanostatic charge discharge (GCD, Supplementary Fig. S11) profiles were obtained, indicating typical capacitive behavior of MXene nanosheets. Remarkably, the linear dependence of the current response on the scan rate was ≤500 V s^−1^ (Fig. 3b), demonstrative of superior electronic and ionic conductivity of MXene microelectrodes, which was further confirmed by a low equivalent series resistance of 0.3 Ω cm^2^ (inset of Fig. 3c). Our M-MSC exhibited a low phase angle of −77.8° at 120 Hz and the cross-over frequency at which the phase angle reached −45° was 2153 Hz (Fig. 3c), close to commercial Al electrolytic capacitors [15]. It is strongly suggestive of the potential of M-MSCs for alternating current-line filtering. Furthermore, our M-MSC delivered satisfactory areal capacitance of 4.1 mF cm^−2^ and competitive volumetric capacitance of 457 F cm^−3^ at 10 mV s^−1^ (Fig. 3d), benefitting from abundant edge planes and superior electrochemical activity of small-sized MXene nanosheets. The areal capacitance could be linearly improved by increasing the film thickness of the microelectrodes while maintaining a constant volumetric capacitance (Supplementary Fig. S12). Our M-MSC showed 83% capacitance retention after 5000 cycles (Supplementary Fig. S13), validating its reliability in actual applications. To extend the working voltage and increase the energy density of a single M-MSC, we further examined the electrochemical performance of the M-MSC in an ionic liquid-gel electrolyte composed of poly (vinylidene fluoride-hexafluoropropylene) and 1-ethyl-3-methylimidazolium tetrafluoroborate (PVDF-HFP-EMIMBF_4_). As observed from CV curves at 0.1–100 V s^−1^ and GCD profiles at 20–500 μA cm^−2^, our M-MSC in PVDF-HFP-EMIMBF_4_ exhibited a wide voltage window of 2.7 V and ultra-high rate capability (Fig. 3e and f, and Supplementary Fig. S14). It is worth noting that the maximal scan rates of our M-MSCs in both aqueous and ionic liquid electrolytes are superior to most reported MXene-based MSCs so far (Fig. 3g) [15,27–29]. This prominent feature of our M-MSCs is attributed to the strong interfacial interaction of small-sized MXene nanosheets and highly conductive current collectors to remarkably boost fast charge transfer and rapid ion transport of the whole device.

To verify the high performance of the M-MSC, a Ragone plot is shown in Fig. 3h. Notably, our M-MSC delivered a volumetric energy density of 2.8 mWh cm^−3^ in PVA/H_2_SO_4_ and 19.2 mWh cm^−3^ in PVDF-HFP-EMIMBF_4_, comparable with lithium thin-film batteries (1–10 mWh cm^−3^) [30] and previously reported MXene-based MSCs [15,27–29,31–33]. In addition, the M-MSC offered an ultra-high power density of 2204 W cm^−3^ (energy density of 0.7 mWh cm^−3^) in PVA/H_2_SO_4_ and 825 W cm^−3^ (energy density of 6.2 mWh cm^−3^) in PVDF-HFP-EMIMBF_4_, which is higher than that of a conventional supercapacitor and even comparable to high-power electrolytic capacitors (10^1^–10^3^ W cm^−3^) [30].

### Integration for ultra-high output voltage

**Figure 4.**
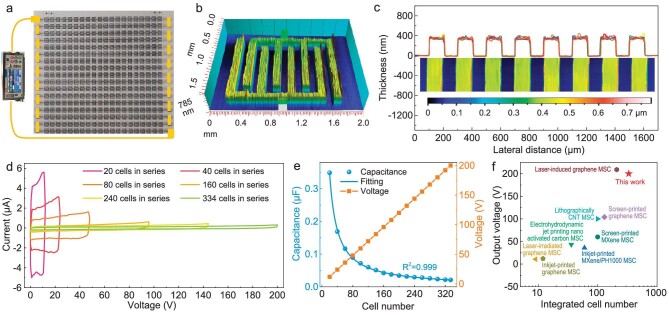
Superior performance uniformity and ultra-high voltage output of M-MIMSCs in PVA/H_2_SO_4_ gel electrolyte. (a) Photograph of M-MIMSCs with 340 cells connected in series. (b) 3D thickness mapping of a selected cell in (a). (c) Thickness profiles of nine M-MSCs at different locations in (a). (d and e) CV curves obtained at 4.8 V s^−1^ (d) and output voltage and capacitance versus cell number (e) of M-MIMSCs containing 20, 40, 80, 160, 240 and 334 cells connected in series. (f) Integrated cell number and output voltage of our M-MIMSCs compared with previously reported integrated MSCs (laser-irradiated graphene MSC [24], inkjet-printed graphene MSC [10], inkjet-printed MXene/PH1000 MSC [14], electrohydrodynamic jet-printing nano-activated carbon MSC [22], screen-printed MXene MSC [13], lithographically CNT MSC [34], screen-printed graphene [11] and laser-induced graphene MSC [23]).

To demonstrate performance uniformity and integration, M-MIMSCs comprising 340 cells with tailored integrated connections were fabricated on a small area (3.5 × 3.5 cm^2^, Fig. 4a). 3D thickness mapping (Fig. 4b) and high-magnification scanning electron microscopy (SEM) imaging (Supplementary Fig. S15) of a selected cell in Fig. 4a presented a flat and continuous surface texture, testifying to the uniformity and nondestructive nature of the spray-printing and lift-off process. Additionally, the thickness profiles of nine independent M-MSCs at different regions in the M-MIMSCs are shown in Fig. 4c, further indicating exceptional uniformity on a larger scale. All the CV curves of our M-MIMSCs connected in series from 20 to 334 cells, obtained at 4.8 V s^−1^ in PVA/H_2_SO_4_ electrolyte, displayed nearly rectangular shapes with typical capacitive behavior and accordingly a step-wise linear increase in the output voltage from 12 to 200 V (Fig. 4d). Such extraordinary tandem capacitive behavior was also demonstrated by GCD profiles with symmetrical triangular shapes, showing linearly increasing output voltage and invariable charge/discharge time (Supplementary Fig. S16). Superior modularization and performance uniformity across each microcell were evidenced by a plot of the capacitance and voltage versus the cell number, which delivered a linear increase in the output voltage and a non-linear decrease in the capacitance with a correlation coefficient close to 1 (Fig. 4e). It is noted that 334 cells and 200 V are the highest integrated cell number and output voltage reported for serially integrated MSCs to date (Fig. 4f) [10,13,14,22–24,34]. Meanwhile, the overall capacitance can be readily enhanced by customizable parallel connections of M-MIMSCs. As shown in Supplementary Fig. S17, 20 M-MSCs connected in series as a cell pack and multiple packs connected in parallel exhibited a simultaneous increase in output current, voltage and capacitance. Therefore, our strategy is highly customizable for producing monolithic integrated microscale energy storage with high output voltage and tailored capacitance to meet the varying customizable requirements in actual scenarios. Additionally, the stretchable M-MIMSCs were fabricated by introducing a honeycomb structure between the microcells on a flexible polyethylene terephthalate substrate (Fig. 1g, Supplementary Fig. S18 and Supplementary Video S2). Notably, the resultant M-MIMSCs could be stably charged and discharged under dynamic stretching to 225% without electrochemical performance degradation, exhibiting excellent mechanical stretchability.

### Integration for ultra-high areal output voltage

**Figure 5.**
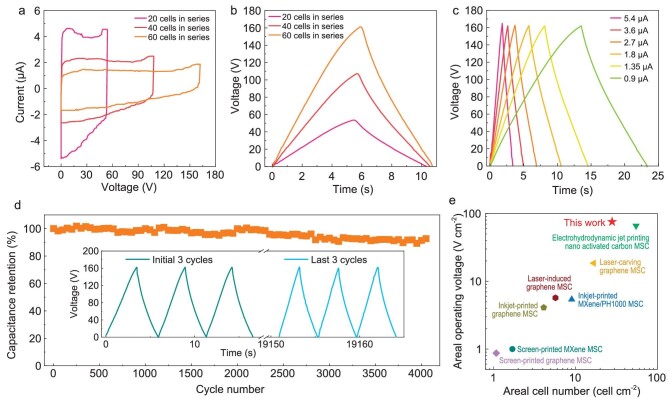
Ultra-high areal output voltage of M-MIMSCs in PVDF-HFP-EMIMBF_4_ gel electrolyte. (a and b) CV curves measured at 11 V s^−1^ (a) and GCD profiles obtained at 1.8 μA (b) of M-MIMSCs containing 20, 40 and 60 cells connected in series. (c and d) GCD profiles measured at different currents (c) and cycling stability for 4000 cycles tested at 2.7 μA (d) of 60 cells connected in series under output voltage of 162 V. Inset in (d) is the GCD profiles during cycles. (e) Areal cell number and areal output voltage comparing our M-MIMSCs with other previously reported integrated MSCs (screen-printed graphene MSC [11], screen-printed MXene MSC [13], inkjet-printed graphene MSC [10], inkjet-printed MXene/PH1000 MSC [14], laser-induced graphene MSC [23], laser-carving graphene MSC [35], electrohydrodynamic jet-printing nano-activated carbon MSC [22]).

To further highlight the customizability and universality of the reliable 3D printing technique, PVDF-HFP-EMIMBF_4_ gel electrolyte was deposited on M-MIMSCs composed of 60 serially-connected microcells. As expected, a series of CV curves and GCD profiles showed outstanding capacitive behavior over the whole voltage range along with a step-wise increasing voltage of ≤162 V (Fig. 5a and b, and Supplementary Fig. S19). They could be stably operated at different scan rates and current densities (Supplementary Fig. S20 and Fig. 5c), delivering a high systemic volumetric energy density of 9.8 mWh cm^−3^. In addition, our M-MIMSCs exhibited 92% capacitance retention over 4000 cycles even at a landmark output voltage of 162 V (Fig. 5d), benefitting from the durability of MXene nanosheets and exceptional performance uniformity of all the microcells. Only 60 serially-connected M-MIMSCs could reach an ultra-high voltage output of 162 V, corresponding to an ultra-high areal output voltage of 75.6 V cm^−2^, outperforming all previously reported integrated MSCs (Fig. 5e), such as the screen-printed graphene MSC (0.87 V cm^−2^) [11], screen-printed MXene MSC (1 V cm^−2^) [13], inkjet-printed graphene MSC (4.1 V cm^−2^) [10], inkjet-printed MXene/PH1000 MSC (5.4 V cm^−2^) [14], laser-induced graphene MSC (5.7 V cm^−2^) [23], laser-carving graphene MSC (18.52 V cm^−2^) [35] and electrohydrodynamic jet-printing nano-activated carbon MSC (65.9 V cm^−2^) [22]. The total output current and discharge time of M-MIMSCs increased proportionally by increasing the number of parallel-connected packs while the output voltage was unchanged at 54 V, resulting in an increase in both the capacitance and the output voltage compared with individual cell (Supplementary Fig. S21).

## CONCLUSION

In summary, we have developed a reliable and universal microfabrication strategy combining high-resolution lithographic patterning and spray-printing techniques with elaborate control of 3D printing to achieve on-chip M-MIMSCs with a high areal-number density (340 cells on 3.5 × 3.5 cm^2^, 28 cells cm^−2^), output voltage of ≤200 V, areal output voltage of 75.6 V cm^−2^, systemic volumetric energy density of 9.8 mWh cm^−3^ and long-term stability. Our strategy is expected to be applicable to other MIMSCs and integrated micro-batteries, which could achieve high modular output capacities. Further architectural design (such as microelectrode configuration) is also possible to improve the space utilization of monolithic micropower sources for compact integration and high-systemic-performance-requiring applications.

## METHODS

The detailed preparation and characteristic methods of materials are available in the online Supplementary file.

## Supplementary Material

nwac271_Supplemental_Files
