# Supplementary material for: Monolithic integrated micro-supercapacitors with ultra-high systemic volumetric performance and areal output voltage
Source: Natl Sci Rev. 2022 Nov 26;10(3):nwac271. doi: 10.1093/nsr/nwac271 (PMC9976746; doi:10.1093/nsr/nwac271)
Supplement: nwac271_Supplemental_Files [file nwac271_supplemental_files.zip › Supplementary data.docx]

**Supplementary Information for *National Science Review***

**Monolithic integrated micro-supercapacitors with ultrahigh systemic volumetric performance and areal output voltage**

Sen Wang^1,+^, Linmei Li^2,+^, Shuanghao Zheng^1,6^, Pratteek Das^1,7^, Xiaoyu Shi^1^, Jiaxin Ma^1,7^, Yu Liu^1^, Yuanyuan Zhu^1^, Yao Lu^2,*^, Zhong-Shuai Wu^1,6,*^and Hui-Ming Cheng^3,4,5,*^

^1^State Key Laboratory of Catalysis, Dalian Institute of Chemical Physics, Chinese Academy of Sciences, Dalian 116023, China;

^2^Department of Biotechnology, Dalian Institute of Chemical Physics, Chinese Academy of Sciences, Dalian 116023, China;

^3^Faculty of Materials Science and Engineering/Institute of Technology for Carbon Neutrality, Shenzhen Institute of Advanced Technology, Chinese Academy of Sciences, Shenzhen 518055, China;

^4^Shenyang National Laboratory for Materials Science, Institute of Metal Research, Chinese Academy of Sciences, Shenyang 110016, China;

^5^Advanced Technology Institute, University of Surrey, Guildford GU2 7XH, UK;

^6^Dalian National Laboratory for Clean Energy, Chinese Academy of Sciences, Dalian 116023, China;

^7^University of Chinese Academy of Sciences, Beijing 100049, China

***Corresponding authors.** E-mails: luyao@dicp.ac.cn; [wuzs@dicp.ac.cn](mailto:wuzs@dicp.ac.cn); cheng@imr.ac.cn

^+^Equally contributed to this work.

**Methods**

**Synthesis of Ti_3_C_2_T_X_ Mxene.** Ti_3_C_2_T_X_ MXene was synthesized from the Ti_3_AlC_2_ by LiF/HCl etchants previously reported [1]. Typically, LiF (0.5 g) was dissolved into 9 M HCl (10 mL) under stirring. Then, Ti_3_AlC_2_ (0.5 g) was slowly added into the above solution, and the resultant mixture was stirred at 35 ^o^C for 24 h. Subsequently, the etched product was washed with deionized water through several rounds of centrifugation (3500 rpm, 5 min) until the pH value reached 6. Afterwards, the obtained Ti_3_C_2_T_X_ sediment was mixed with deionized water (10 mL) and then subjected to vigorous shaking by using a vortex machine. Successively, the obtained dispersion was centrifuged (1500 rpm, 1 h) and then the collected sediment was subjected to further high speed centrifugation (3500 rpm, 1 h). After the resultant sediment was redispersed in deionized water by shaking vigorously, Ti_3_C_2_T_X_ MXene aqueous dispersion with a concentration of 1 mg mL^-1^ was achieved. To obtain smaller flake sizes for further usage, MXene suspension was sonicated for 15 min by using a tip sonicator while stirring in an ice bath.

**Fabrication of gel electrolyte.** PVDF-HFP-EMIMBF_4_ gel electrolyte was prepared according to the following steps. First, PVDF-HFP (0.2 g) was dissolved in acetone (2 mL) under stirring to form a transparent solution. Then, EMIMBF_4_ (1.8 g) was added dropwise into the above solution under continuous stirring for 2 h. After being solidified in vacuum drying oven at 70 °C for 8 h, PVDF-HFP-EMIMBF_4_ gel electrolyte was obtained. The PVA/H_2_SO_4_ gel electrolyte was prepared by mixing 10 g H_2_SO_4_ and 7.5 g PVA (Mw=67000) in 50 mL deionized water and heated up to 80 °C for 2 h under vigorous stirring [2].

**Preparation of M-MIMSCs.** First, the substrates (e.g., Si, glass, and flexible polyethylene terephthalate) were cleaned by sequential bath in ethanol and deionized water for 1 h. Next, a thin photoresist (AZ4620) was coated onto the target substrate and then exposed to ultraviolet light through a photomask, forming the pre-designed patterns of metal current collectors. After development in AZMIF-300 solution, the exposed regions of photoresist were dissolved, leaving behind the pre-designed pattern. Next, a thin layer of Au/Ti was sputtered over the substrate followed by lift-off procedure enabled by immersion in acetone, forming the current collectors for electrodes, electrical connections between adjacent devices and for performing external measurements. Interdigitated fingers have a typical width of 100 µm, length of 1000 µm, interspacing of 100 µm, and cell-to-cell spacing of 600 µm. Then, another photoresist layer (S1805) with microelectrode pattern on Au/Ti metal collectors was obtained by the same process. Subsequently, MXene microelectrodes were achieved by spray printing of 1 mg mL^-1^ MXene dispersion by an automatic spraying equipment, followed by lift-off in acetone assisted by ultrasonic treatment*.* By controlling the volume of spray-printed dispersion, the thickness of MXene microelectrodes could be varied. Finally, the M-MIMSCs was obtained by 3D printing quasi-solid-state gel electrolyte allocated onto the cell using an extrusion-based 3D printer.

**3D printing gel electrolyte.** 3D printing was conducted on a benchtop robotic dispenser (ZZ-221, ZhongZhi Automation Co., Ltd.) and an air-powered fluid dispenser (JND983A) was adopted to pressurize the barrel and control the ink flow rate. First, desired printing path was designed using AutoCAD software and transferred to the robotic dispenser to control the motion of nozzles. Second, the prepared gel electrolyte was housed in 30 mL syringe ready to be printed. Third, 3D printing parameters, such as extrusion nozzle’s inner diameter, printing speed and extrusion pressure were tuned for precisely depositing gel electrolytes and enabling the electrochemical isolation of each cell, avoiding their incomplete or uneven coverage over microelectrodes, and contact with each other. In the appropriate rectangle printing path, the inner diameter of the extrusion needles was 210 µm, printing speed was 4 mm s^-1^, and extrusion pressure was 25 psi for PVA/H_2_SO_4_ gel electrolyte. The inner diameter of the extrusion needles was 210 µm, printing speed was 1 mm s^-1^, and extrusion pressure was 10 psi for PVDF-HFP-EMIMBF_4_ gel electrolyte.

**Electrochemical characterization of M-MSC and M-MIMSCs.** The electrochemical performance of single M-MSC was carried out by CV curves from 0.01 to 500 V s^-1^, GCD measurements from 10 to 500 μA cm^-2^ and electrochemical impendence spectroscopy recorded in the frequency range of 0.01 Hz ∼ 100 kHz with an alternating current amplitude of 5 mV on an electrochemical workstation (CHI 760E). The electrochemical performance of high-voltage M-MIMSCs was carried out by CV curves (scanning step from 0.5 to 3 V, test delay of 0.005 s), and GCD profiles from 0.9 to 5.4 μA on Keithley 2450.

Based on CV curves, the capacitance values were calculated according to the following equations (1):

$C_{device}=\frac{1}{\nu(V_{f}-V_{i})}\int_{V_{f}}^{V_{i}} I(V)dV$ (1)

$C_{electrode}=4C_{device}$ (2)

Where $C_{device}$ is the capacitance based on the device, *C_electrode_* is the capacitance contribution mainly from MXene microelectrodes, *ν* is the scan rate (V s^-1^), *V_f_* and *V_i_* are the integration potential limits of the voltammetric curve, and *I (V)* is the voltammetric discharge current (A).

Based on GCD profiles, the capacitance values were calculated in term of the following equations:

$C_{device}=\frac{It}{\Delta U}$ (3)

Where *I* is the discharge current from GCD profiles (A), *t* is the discharge time (s). Δ𝑉 is the discharge voltage window (V).

Specific capacitance was calculated in term of the area or volume of the device according to the following formula:

$C_{A}=\frac{C}{A}$ (4)

$C_{V}=\frac{C}{V}$ (5)

Where *C_A_* (mF cm^-2^) and *C_V_* (F cm^-3^) refers to the areal capacitance and volumetric capacitance, respectively. *A_electrode_* and *V_electrode_* is the total area (cm^2^) and volume (cm^3^) of the microelectrodes, respectively. *A_device_* is the entire projected surface area of the device, including the area of microelectrodes and the interspaces between them. *V_device_* was calculated by taking into account the whole volume of the device, including the volume of MXene microelectrodes and the interspaces between the microelectrodes.

The energy density and power density were calculated based on the volumetric capacitance of the device (*C_V,device_*) measured under the same dynamic condition from the discharge curves of cyclic voltammetry. The energy density of the device was obtained from the equation:

$E=\frac{1}{2}\times C_{V,device}\times\frac{\Delta V^{2}}{3600}$ (6)

where *E* is the energy density (Wh cm^-3^), $C_{V,device}$ is the volumetric capacitance obtained from equation (5) and *ΔV* is the discharge voltage (V).

The power density of the device was calculated from the formula:

$P=\frac{E}{\Delta t}\times3600$ (7)

where *P* is the power density (W cm^-3^), and *Δt* is the discharge time (in seconds).


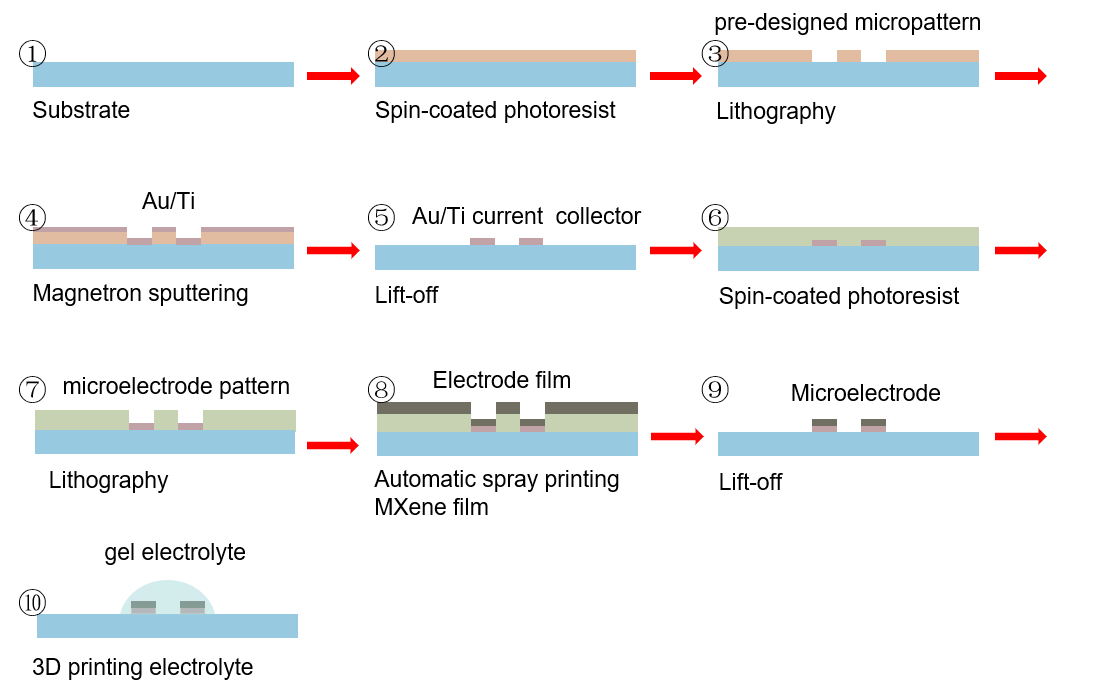


**Fig. S1.** The detailed processes for the step-by-step microfabrication of M-MIMSCs.


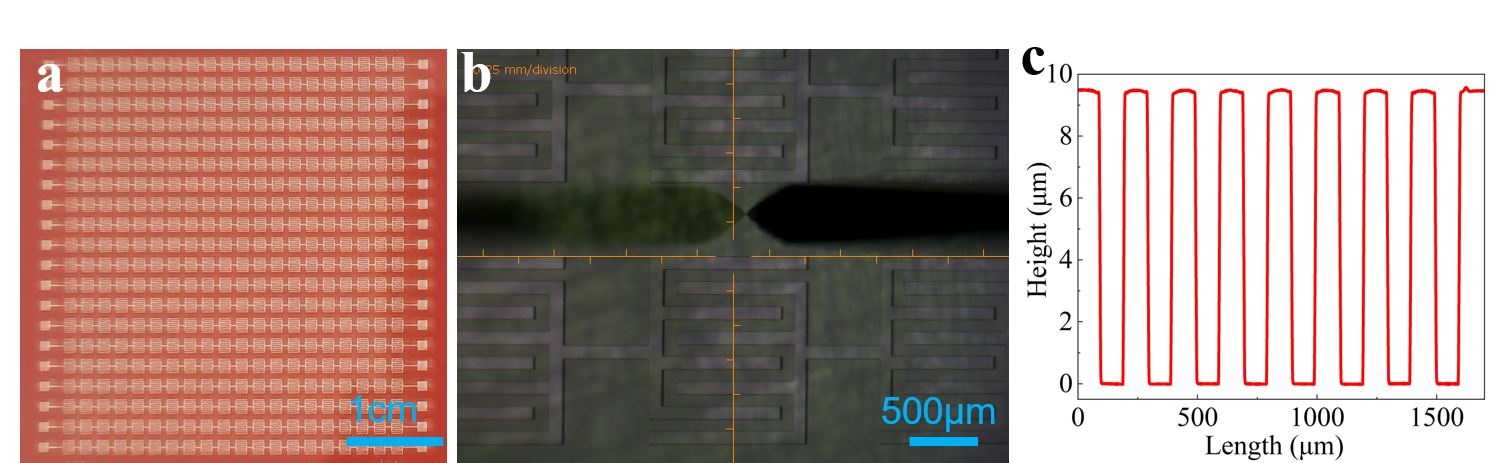


**Fig. S2.** Characterization of AZ4620 photoresist layer with pre-designed metal current pattern on glass substrate, corresponding to the step 3 in Fig. S1. (a) Digital photograph. (b) Optical microscope photograph. (c) Height profile.


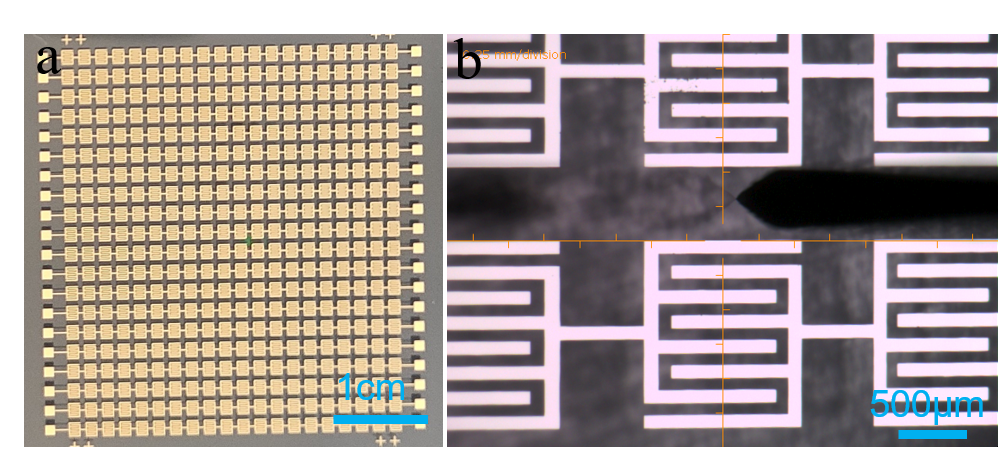


**Fig. S3.** Characterization of Au/Ti current collectors for M-MIMSCs on glass substrate, corresponding to the step 5 in Fig. S1. (a) Digital photograph, and (b) optical microscope photograph.


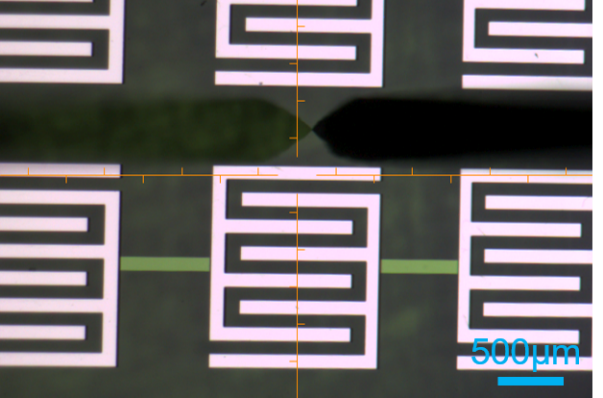


**Fig. S4.** Optical microscope photograph of S1805 photoresist layer with microelectrodes pattern on Au/Ti current collectors, corresponding to the step 7 in Fig. S1.


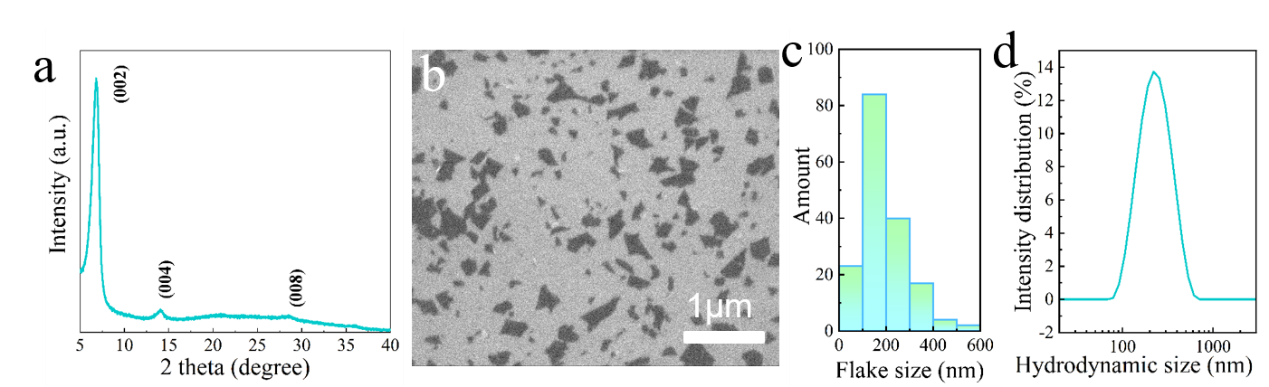


**Fig. S5.** Characterization of nano-sized MXene nanosheets. (a) XRD pattern, (b) SEM image, (c) flake size distribution, and (d) dynamic light scattering intensity distribution.


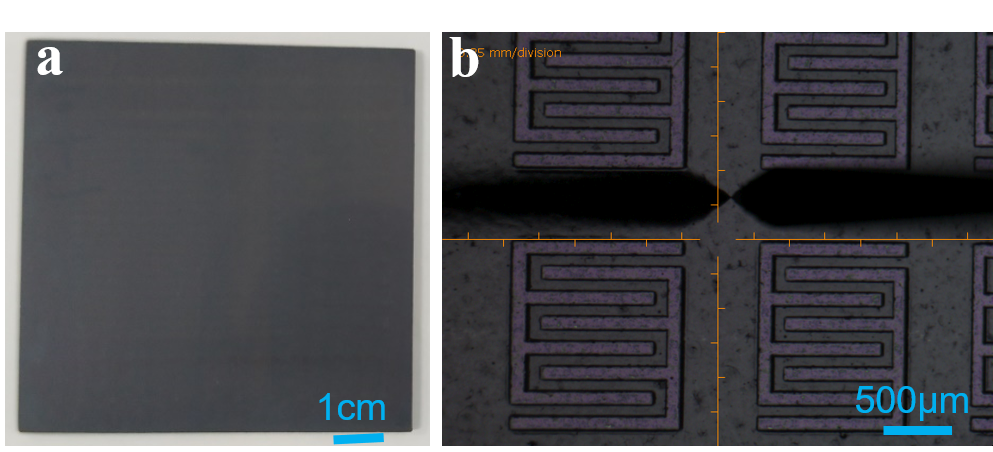


**Fig. S6.** Characterization of MXene film coated on patterned photoresist, corresponding to the step 8 in Fig. S1. (a) Digital photograph, and (b) optical microscope photograph.


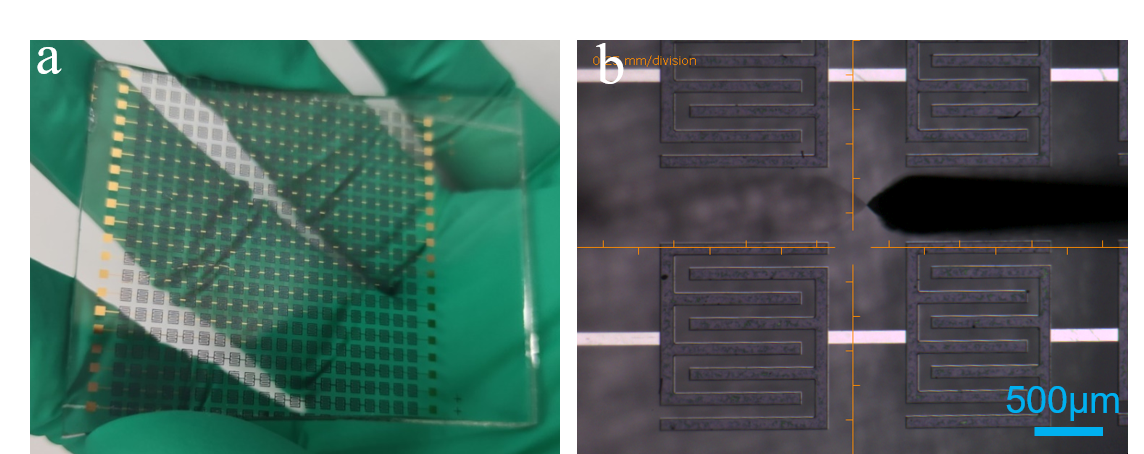


**Fig. S7.** Characterization of M-MIMSCs on glass substrate, corresponding to the step 9 in Fig. S1. (a) Digital photograph, and (b) optical microscope photograph.


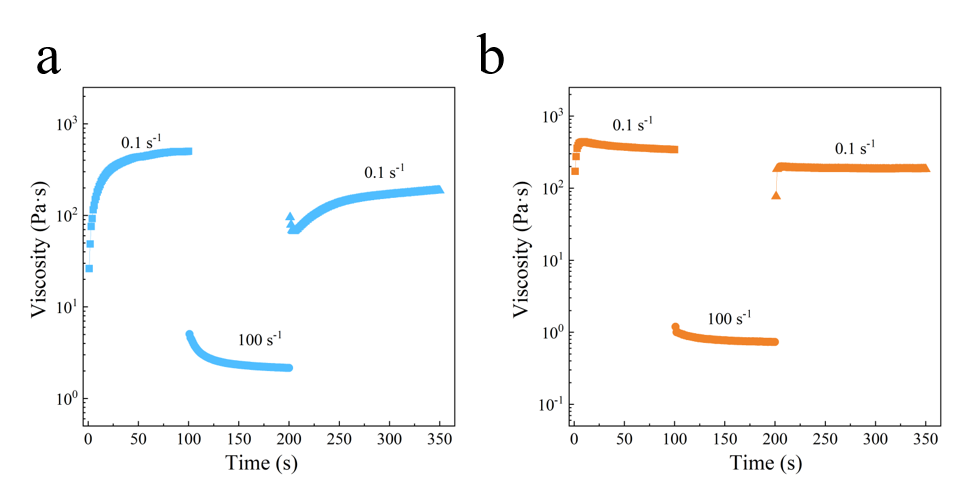


**Fig. S8.** Rheological behavior during the extrusion printing process of gel electrolyte inks. (a) PVA/H_2_SO_4_ gel electrolyte ink, and (b) PVDF-HFP-EMIMBF_4_ gel electrolyte ink.


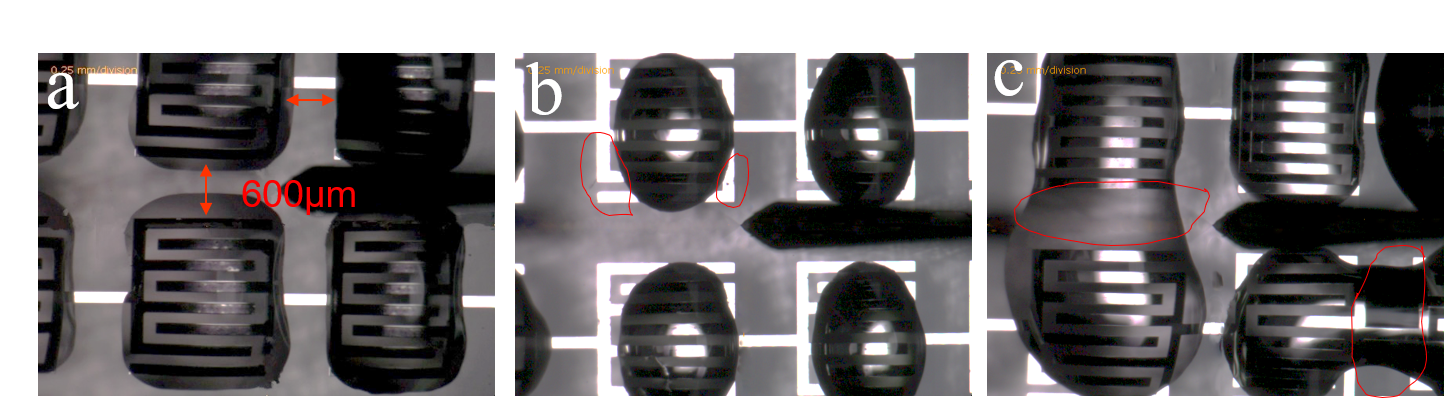


**Fig. S9**. Optical microscope photographs of 3D printing gel electrolyte under rectangle printing path, corresponding to the step 10 in Fig. S1. (a) Gel electrolyte accurately deposited on each cell with the adjacent interval of 600 μm under optimal condition. (b) Gel electrolyte incomplete coverage or uneven coverage the microcells, resulting in incomplete utilization or poor uniformity between multiple cells, and (c) gel electrolyte contacted between adjacent microcells, resulting in failure electrochemical isolation under unsuitable conditions.


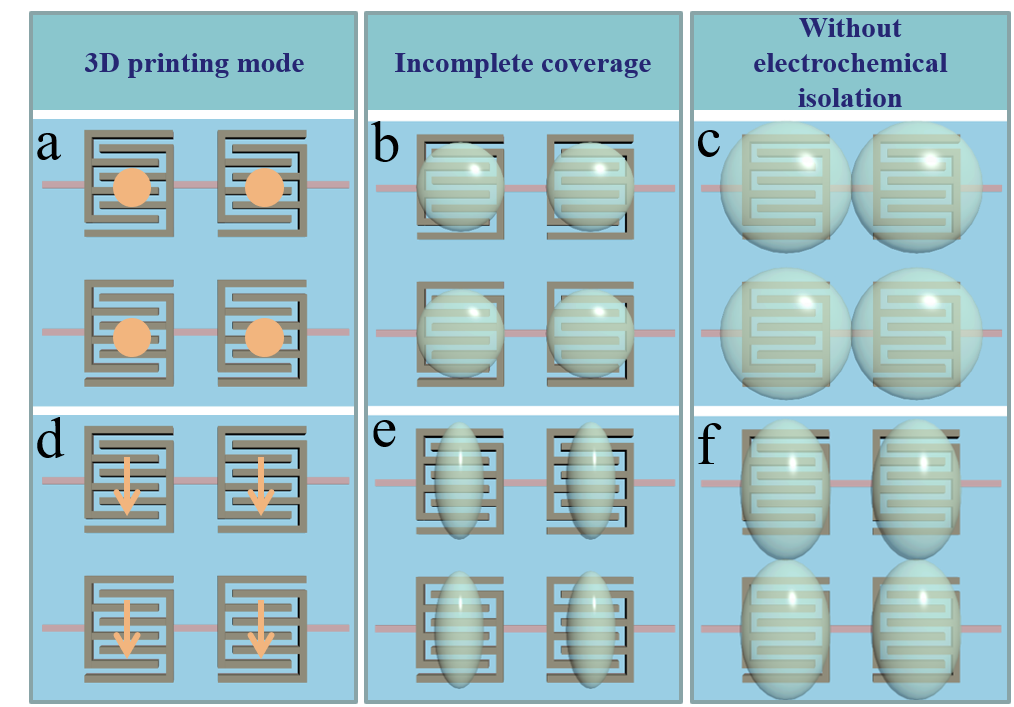


**Fig. S10**. Schematic of 3D printing gel electrolyte under different printing modes. (a-c) Dot 3D printing mode, (d-f) line segment 3D printing mode.

No matter how to adjust the printing parameters in an inappropriate printing mode, the electrolyte cannot achieve complete coverage of the cells and guarantee electrochemical isolation, simultaneously.


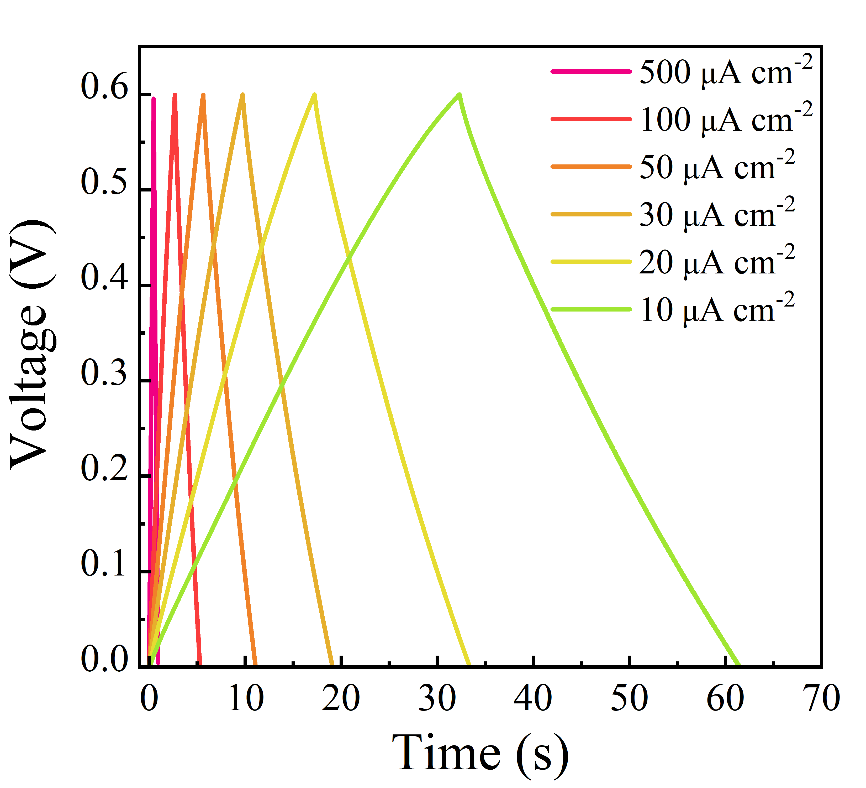


**Fig. S11.** GCD profiles of M-MSC tested at current densities of 10-500 μA cm^-2^ in PVA/H_2_SO_4_ electrolyte.

**
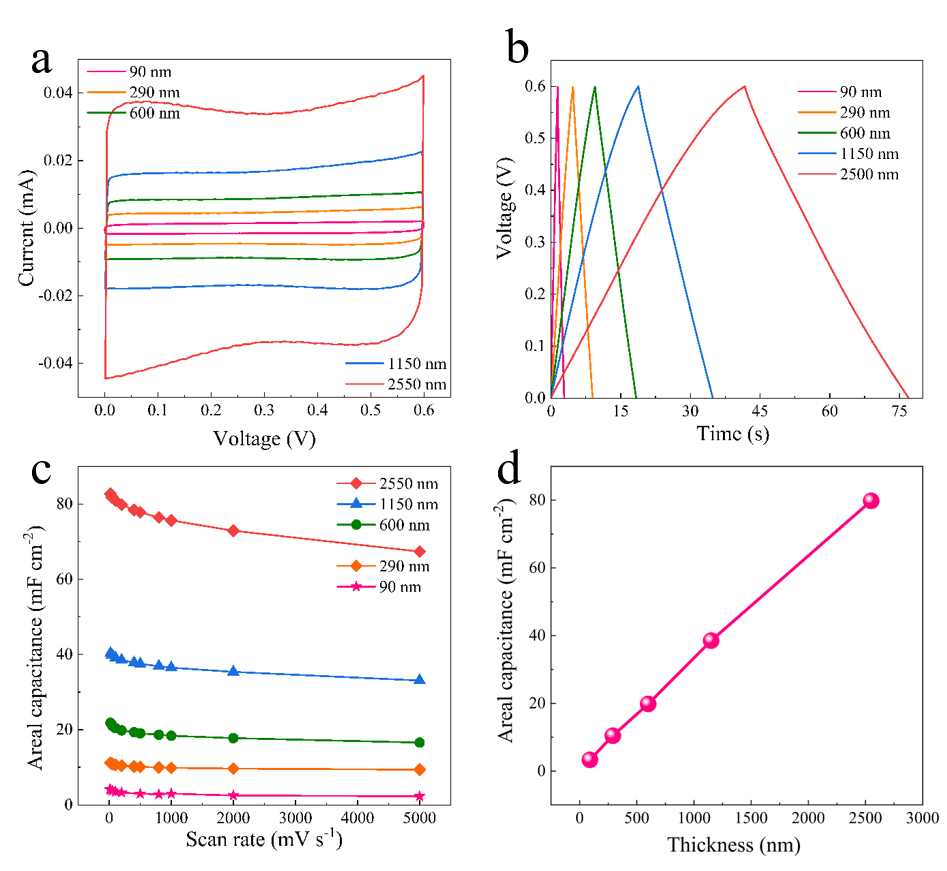
**

**Fig. S12.** Electrochemical performance of M-MSCs based on different film thickness in PVA/H_2_SO_4_ electrolyte. (a) CV curves obtained at scan rate of 200 mV s^-1^, (b) GCD profiles tested at current of 3.2 μA, and (c) the plot of areal capacitance of M-MSCs based on different film thickness. (d) Areal capacitance versus film thickness of M-MSCs obtained at 200 mV s^-1^.


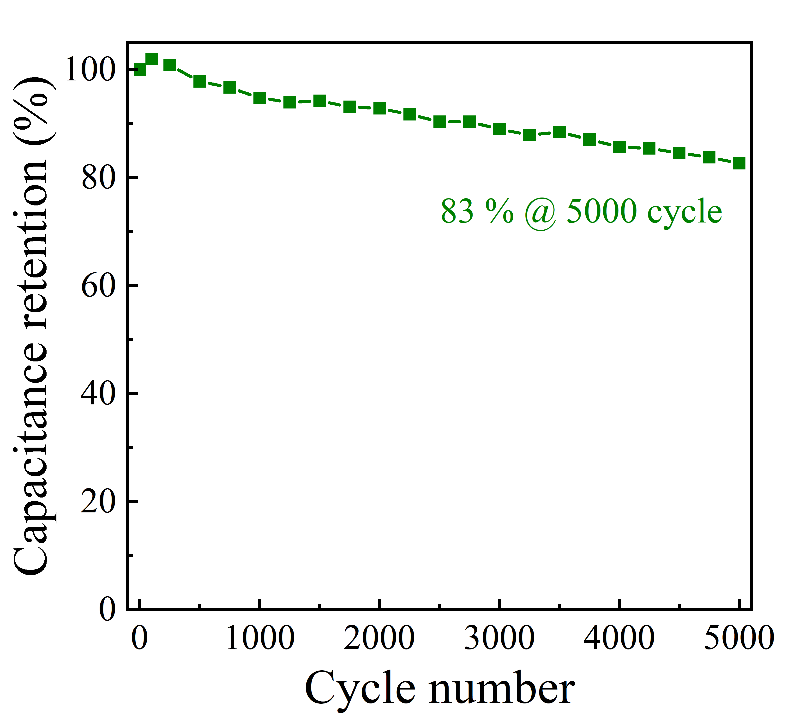


**Fig. S13.** Cycling stability of M-MSC at a current density of 200 μA cm^-2^ in PVA/H_2_SO_4_ electrolyte.


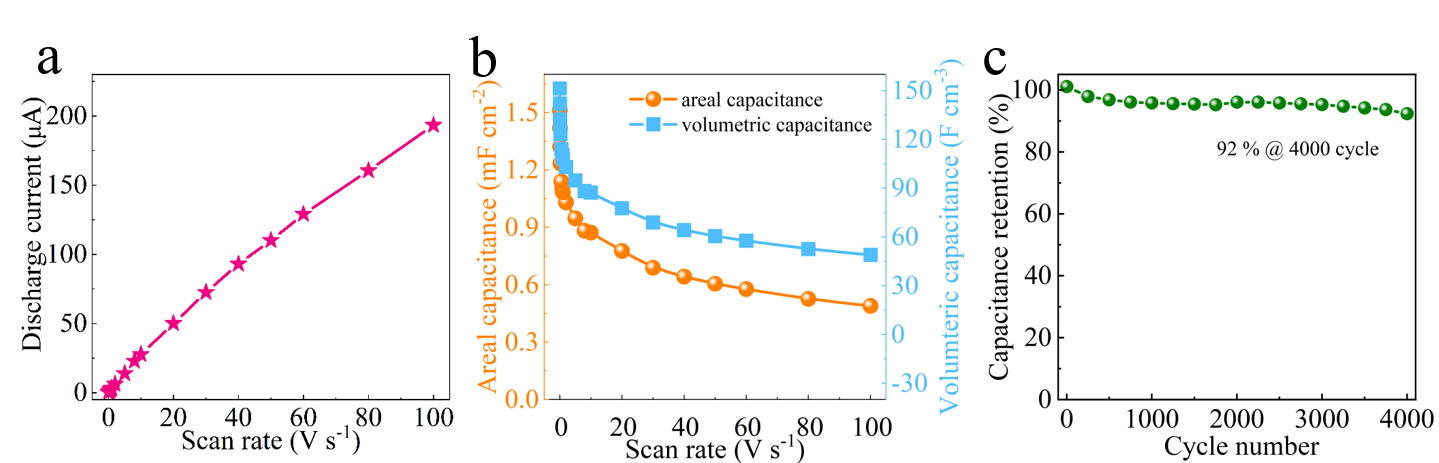


**Fig. S14.** Electrochemical performance of M-MSC in PVDF-HFP-EMIMBF_4_ electrolyte. (a) Discharge current as a function of scan rate, (b) change of the volumetric capacitance and areal capacitance versus scan rate, and (c) cycling stability at a current density of 100 μA cm^-2^.


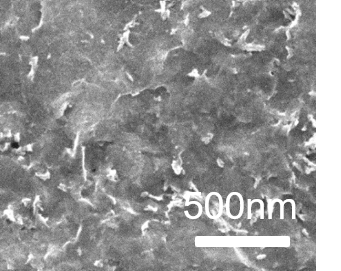


**Fig. S15.** High-magnification top view SEM image of MXene microelectrode.


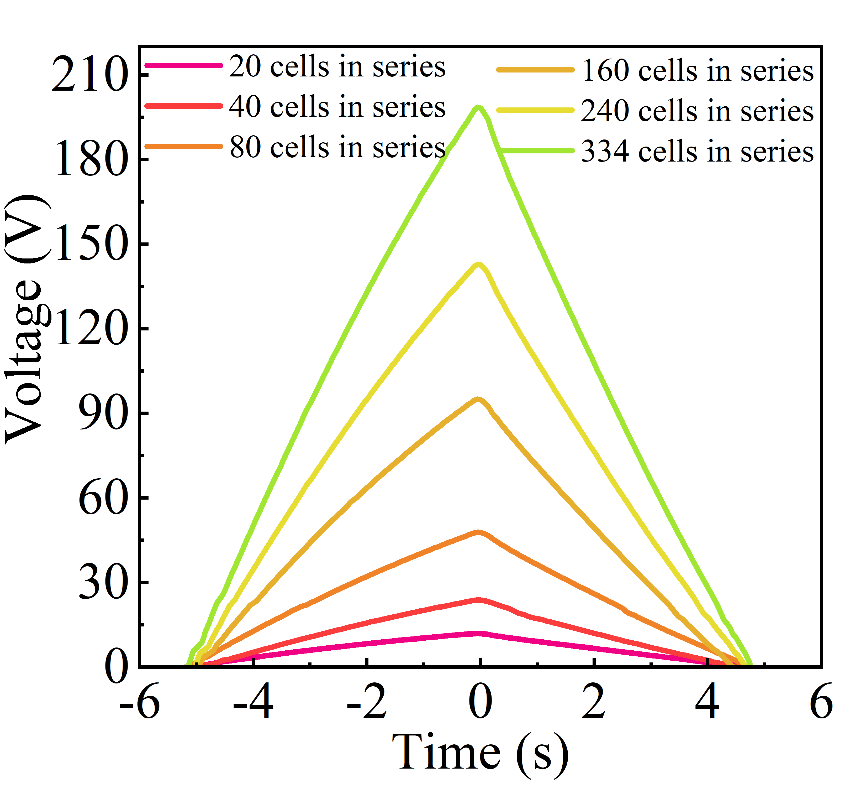


**Fig. S16.** GCD profiles at 0.9 μA of M-MIMSCs containing 20, 40, 80, 160, 240 and 334 cells connected in series in PVA/H_2_SO_4_ electrolyte.


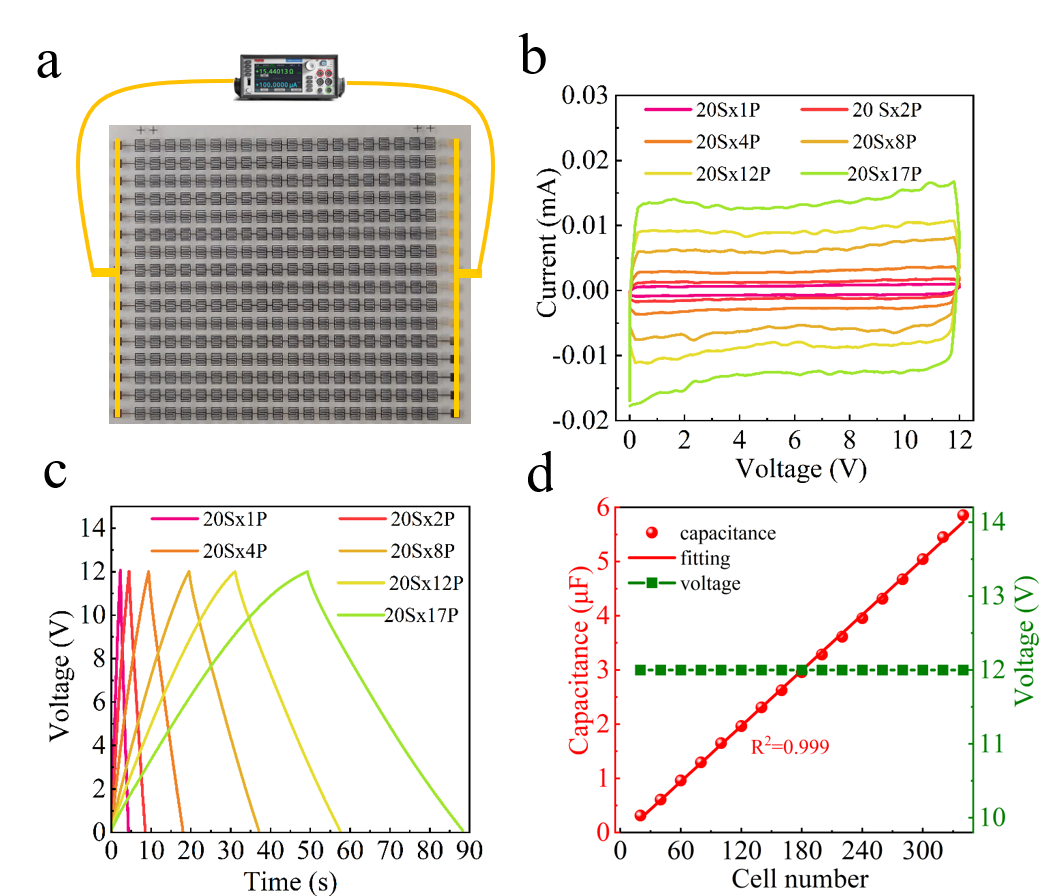


**Fig. S17.** Tailored capacitance and voltage of M-IMSCs in PVA/H_2_SO_4_ electrolyte. (a) Photography of M-MIMSCs with 340 cells, 20 serially-connected M-MSCs as a cell pack (20S) and then multiple packs connected in parallel (20S × *y*P, in which y represent the number of cell packs connected in parallel). (b) CV curves obtained at 4.8 V s^-1^, (c) GCD profiles at 1.8 μA, and (d) output voltage and capacitance as functions of cell number calculated from GCD profiles of M-MIMSCs containing 20, 40, 80, 160, 240 and 340 cells.


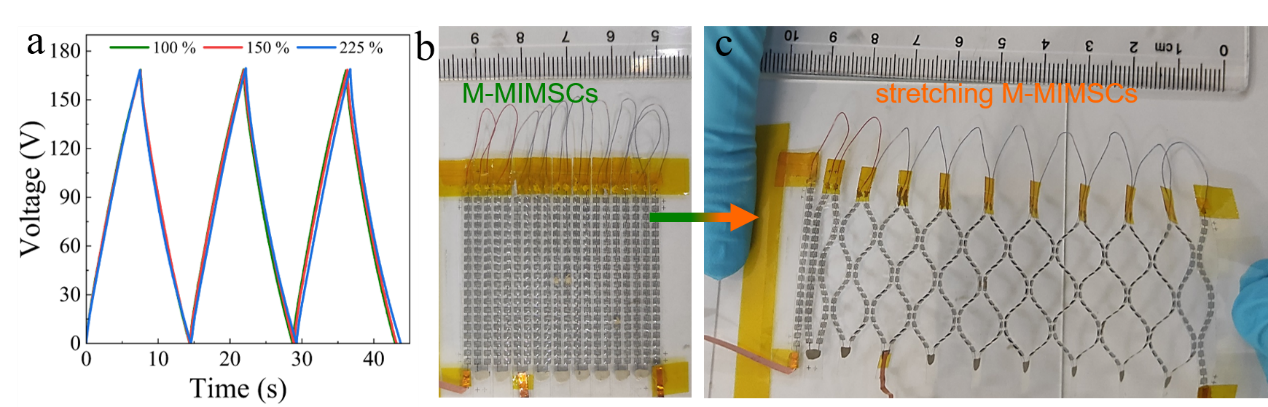


**Fig. S18.** (a) GCD profiles measured at 0.9 μA of M-MIMSCs (thickness of 200 nm) containing 280 cells connected in series under different stretching states in PVA/H_2_SO_4_ electrolyte. (b,c) Digital images of M-MIMSCs under (b) normal and (c) stretching states.


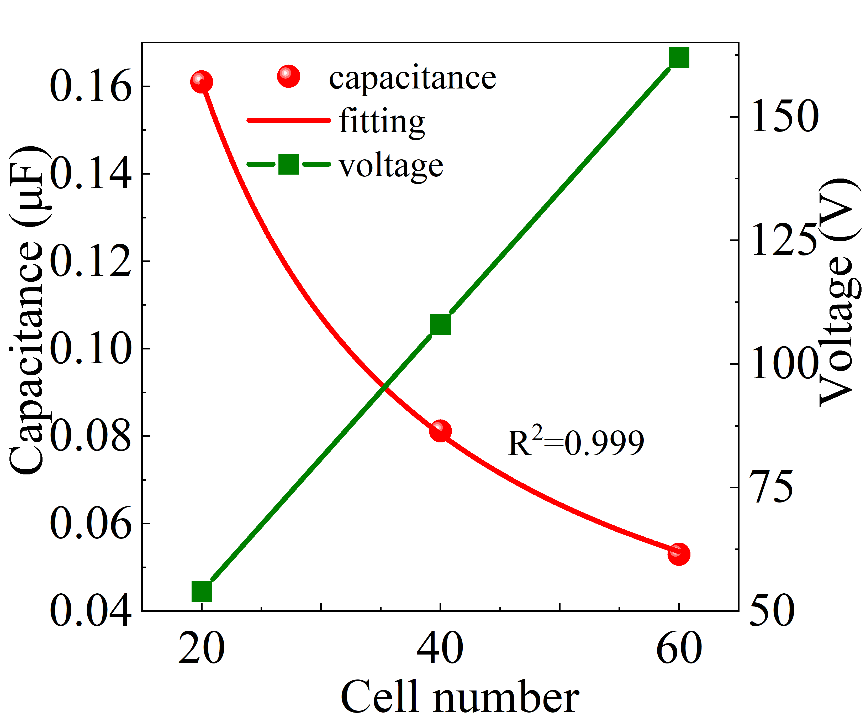


**Fig. S19.** Output voltage and capacitance as functions of cell number calculated from GCD profiles of M-MIMSCs containing 20, 40 and 60 cells connected in series in PVDF-HFP-EMIMBF_4_ electrolyte.


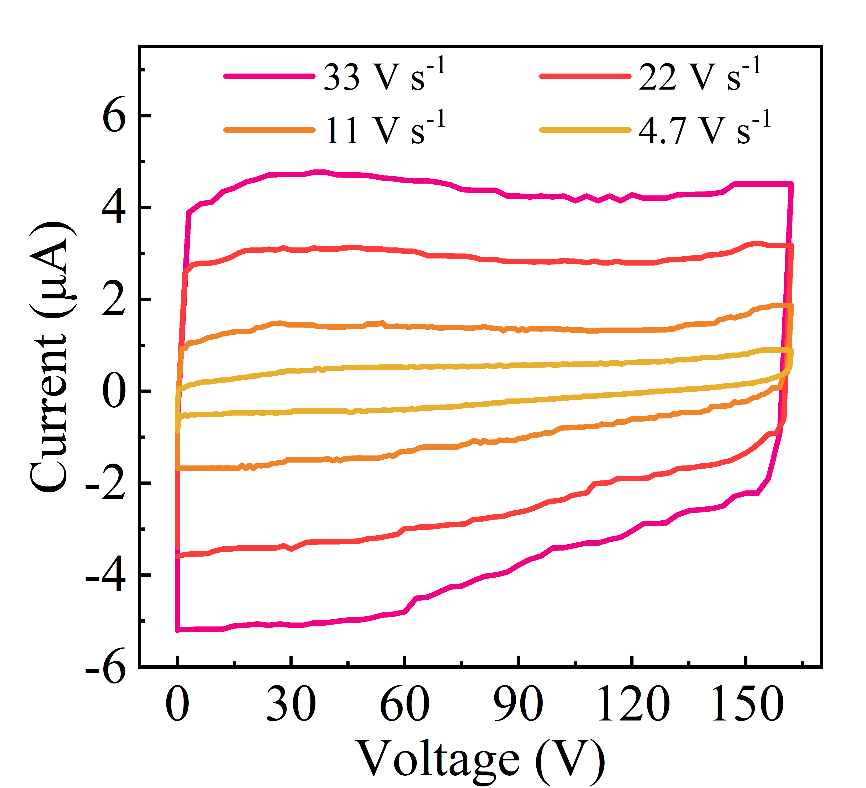


**Fig. S20.** CV curves obtained at different scan rates of 4.7-33 V s^-1^ of M-MIMSCs containing 60 serially-connected cells in PVDF-HFP-EMIMBF_4_ electrolyte.


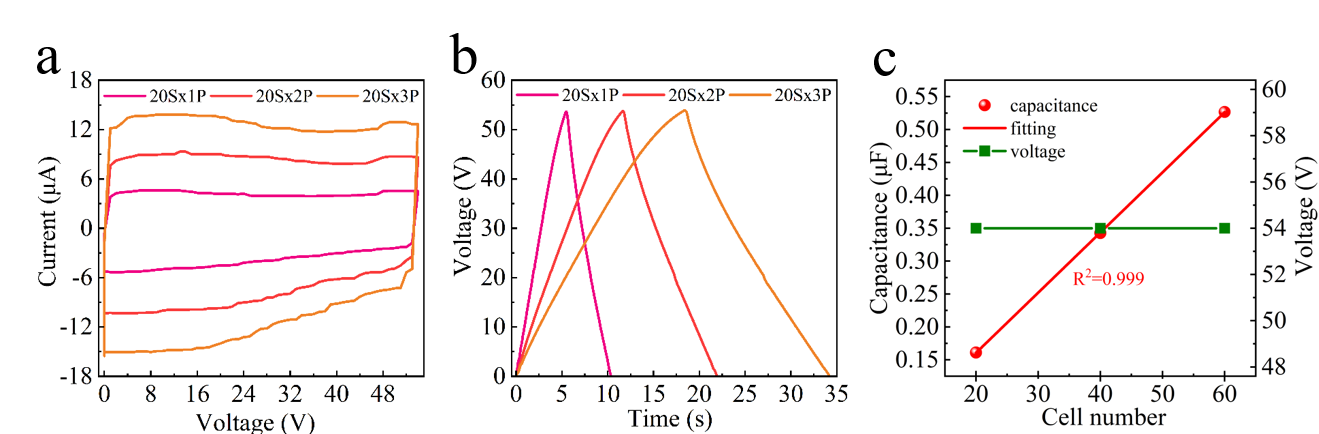


**Fig. S21.** Electrochemical performance of M-MIMSCs with tailored capacitance and voltage in PVDF-HFP-EMIMBF_4_ electrolyte. (a) CV curves obtained at 11 V s^-1^, (b) GCD curves at 1.8 μA, and (c) output voltage and capacitance as functions of cell number calculated from GCD profiles of M-MIMSCs containing 1, 2 and 3 packs (20 M-MSCs connected in series as a cell pack) connected in parallel.

**References**

1. Zheng, S, Zhang, C, Zhou, F, et al. Ionic liquid pre-intercalated MXene films for ionogel-based flexible micro-supercapacitors with high volumetric energy density. J Mater Chem A 2019; **7**: 9478-85.

2. Wu, Z, Parvez, K, Feng, X, et al. Graphene-based in-plane micro-supercapacitors with high power and energy densities. Nat Commun 2013; **4**: 2487.
